# Potential predictive factors for successful referral from specialist mental-health services to less intensive treatment: A concept mapping study

**DOI:** 10.1371/journal.pone.0199668

**Published:** 2018-06-26

**Authors:** Thijs Beckers, Bauke Koekkoek, Giel Hutschemaekers, Bea Tiemens

**Affiliations:** 1 Research Group Social Psychiatry and Mental Health Nursing, Hogeschool van Arnhem Nijmegen (University of Applied Sciences), Nijmegen, The Netherlands; 2 Primary Mental Healthcare, MET ggz, Roermond, The Netherlands; 3 Clinical Psychology, Behavioral Science Institute, Radboud University, Nijmegen, The Netherlands; 4 Pro Persona Research Institute, Wolfheze, The Netherlands; 5 Indigo Service Organization, Utrecht, The Netherlands; Centre for Addiction and Mental Health, CANADA

## Abstract

Referring patients from specialist mental-health services (provided by multiple healthcare service providers and aimed at relieving symptoms of mental illness) to less intensive care (provided by a nurse or psychologist in cooperation with a general practitioner and aimed at improving quality of life) is feasible from the perspective of patients, service providers, and mental-health services. However, it is unclear which patients are most suitable for referral to less intensive care. In this study, we used concept mapping to identify factors that might determine whether a referral from specialist mental services to less intensive care might be successful. Participants (N = 34) were recruited from different parts of the Netherlands and included general practitioners, peer workers, community mental-health nurses, and social workers from several services who were based in different neighborhoods. The participants generated 54 statements (31 after clean-up), which were sorted into five clusters and rated on their expected ability to predict successful referral. Ordered from highest to lowest on expected predictive value, the clusters of factors were: Patient characteristics, patients’ informal support system, patients’ social situation, organization of services, and service provider related factors. The ordering was the same for all of the service providers, except that general practitioners expected the organization of services to be the most predictive. The ordering of the clusters is mostly consistent with existing knowledge about recovery during mental healthcare. In order to further improve the number of successful referrals from specialist mental-health services to less intensive care, a prospective prediction study is needed.

## Introduction

Because of mental illness, some patients are seriously limited in their daily functioning, and they might need ongoing treatment for several years. Such patients are considered to have a severe mental illness (SMI)[1], and they have traditionally been treated in specialist mental-health services (SMHS). SMHS provides treatment by teams of multiple healthcare service providers with different backgrounds, e.g., specialized nurses, social workers, psychiatrists, and psychologists; the treatment is typically aimed at relieving the symptoms of mental illness. In the last decade, patients with a SMI, mainly those who have received several years of treatment in SMHS, have increasingly received a form of less intensive treatment.

In the Netherlands, there are different options for less intensive treatment. With the first option, a specialized nurse or a psychologist from a basic mental health service provides care in cooperation with a general practitioner. With the second option, a nurse provides care under direct supervision of the general practitioner. Both of these less intensive treatment options are aimed at improving the patient’s quality of life more than relieving the symptoms of the mental illness. Which patients with SMI need long-term care from SMHS and which patients might be treated in less intensive treatment—and at what point in the treatment—has been an ongoing discussion for more than a decade [2, 3]. A variety of healthcare service providers are involved in the treatment of patients with a SMI, e.g., general practitioners, mental-health nurses, psychologists, psychiatrists, and social workers, and the different kinds of service providers differ in their views about which patients can successfully be referred from SMHS to less intensive treatment.

Roughly, two perspectives can be identified when the matter of referring patients with severe mental illness from SMHS to less intensive treatment is being discussed: The patient’s perspective and the societal-economic perspective. From the patient’s perspective, it might be attractive to discontinue receiving specialized services, because the high level of care provided in SMHS might hamper the person’s recovery and personal responsibility. This might happen, for instance, through a high frequency of contact, an ever-present safety net of specialized service providers, and an environment in which symptomatic recovery (i.e., becoming symptom-free) are often more valued than patients’ personal recovery (being able to adapt their attitudes, values, feelings, goals, skills, and roles to meet the demands of a new situation) [4,5]. According to the *good-enough level* model, continuation of treatment does not necessarily improve patients’ outcomes [6–8], but what is “good enough” is difficult to establish. There are indications that a timely referral to less intensive treatment might support the process of recovery and facilitate assuming new roles, for instance, of spouse, parent, friend, or employee [9].

From the societal and economic perspective, referral of people from SMHS to less intensive treatment might also be desirable. SMHS is more expensive than other treatments, but it is most effective for people with complex mental-health problems. When people who genuinely need it but cannot gain access to SMHS because of the large number of people with less intense needs who are already being treated in these services, both the individuals who are unable to gain access and society at large might suffer the consequences [2, 3, 10]. People with strong needs who are receiving limited care or none at all might cause harm to themselves or others, might come into contact with the police, or might make heavy use of crisis services [11]. To prevent these personal, societal, and economic burdens from happening, specialized services could focus on offering a high level of service to the people who most need it. This might be realized, for example, by referring people with less intense needs to less intensive treatment.

Healthcare service providers who are involved in the care for patients with SMI report that they lack the skills necessary to decide which patients should be referred to less intensive treatment. Because they have difficulty deciding which patients to refer to less intensive treatment and realizing that a wrong decision increases the risk of patients’ relapsing and experiencing a crisis [12], healthcare service providers are reluctant to refer patients to less intensive treatment. Thus, this deficit in service providers’ knowledge decreases the number of referrals to less intensive treatment, and it causes SMHS to become congested and inaccessible to new patients. More information about how best to refer patients with SMI to less intensive treatment is needed. Specifically, clear-cut criteria are needed for predicting successful referrals from SMHS to less intensive treatment. To fill this gap, in this paper we report the outcome of a concept-mapping study of factors that might be useful when deciding which patients should be referred from SMHS to less intensive care and at what point in their treatment such referrals should be made.

## Method

Concept mapping is a structured methodology developed to address complex problems in (for example) healthcare [13]. The concept mapping method is designed to integrate qualitative input from different sources, using rigorous multivariate data analysis for clarification of such qualitative data. The method results in visual maps displaying the consensus of the participants, usable for planning, developing, or evaluating health policy. The concept mapping methodology was chosen for this study because it is an efficient way to explicate, structure and prioritize tacit knowledge about topics on which the research literature is inconclusive. In this study, we used it to explore the clusters of factors that are expected to predict successful referrals, to establish the coherence of these factors, and to prioritize them [14].

### Participants

Participants were recruited from four groups of healthcare service providers who work with patients suffering from severe mental illness: general practitioners, peer workers, community mental-health nurses working in primary care, and neighborhood-based social workers. Service providers who were working in SMHS were not included in the sample, because tacit knowledge about SMHS has been explicated in earlier research. The participants were recruited by the researchers from health services with which the researchers were affiliated with and from the healthcare service providers these health services collaborate with (e.g. general practitioner, community mental health nurses). The involved health services are typical for the Netherlands, each providing several types of mental health services in a specific Dutch region. They were sampled from different parts of the Netherlands. In order to maintain diversity, a maximum of 50% of the participants in each group of service providers was sampled from each mental-health catchment area. Characteristics of the participants were collected at the sorting and rating stage.

#### Data collection and analysis

We used the customary five steps in the concept mapping method [14]: (1) preparation, (2) generation and clean-up of statements, (3) structuring the statements, (4) analysis, and (5) interpretation of the maps. The Global MAX^™^ software from Concept Systems^®^ was used for data collection and analysis. Data was collected in Dutch, as this is the leading language in the area of the data collection (the Netherlands). All translations were primarily done by the first author and were checked against the original text by the other authors. A native English speaker checked the translations for clarity, after which the authors evaluated the suggestions of the native speaker.

### Preparation

The procedure began with deciding how the participants would be recruited (see description above) and how the focus prompt would be formulated. The prompt was a one-sentence question that this study was designed to answer.

### Generating statements

Using the Global MAX software, the participants selected a time within a three-week period that was convenient for them. Participants could see the statements that their peers had made, and they could add additional statements. This procedure differs from the traditional procedure in which statements are generated in one meeting at which participants can react to one another. After the statements had been generated, the statements were cleaned up. This clean-up consisted of removing duplicate statements and checking for confusing language, errors in language, and for statements that contained more than one idea. The clean-up procedure was reviewed by two healthcare service providers that participated in the study.

### Structuring the statements

The statements were structured by asking the participants to sort and rate the statements according to their own opinions, and to do so within a period of four weeks. Participants sorted the statements by categorizing them according to the meaning or theme that each statement conveyed. This was done on a computer by placing virtual cards on which the statements appeared on virtual piles on a virtual desktop. The participants were not allowed to exclude statements or to create ambiguous categories such as *other*. There was no limit to the number of categories that participants could create. After the statements had been sorted, the participants were asked to rate each statement on two 7-point Likert scales. One scale was *importance* (ranging from ‘very unimportant’ to ‘very important’), with the instruction “To what extent are the following statements important for a successful referral from SMHS to less intensive care?”. The other scale was for *predictive value* (ranging from ‘not predictive’ to ‘very predictive’), with the instruction “To what extent are the following statements predictive for a successful referral from SMHS to less intensive care?”.

### Analysis

Multi-dimensional scaling was used to aggregate participants’ structuring of the statements onto locations on a concept map. We used hierarchical cluster analysis to place the statements into groups and to create boundaries around the groups of statements [14]. A stress value was also calculated; this is a goodness-of-fit indicator between a given set of dissimilarities as input and the resultant distances in a diagram [15]. We used 0.39 as the upper limit of the stress value for a reliable concept mapping study [16]. The means of the statements and clusters were then used to indicate their relative priority [14]. Each cluster was given a number for ease of use (ordered from most predictive to least predictive).

### Interpretation of the concept map

A procedure that Rosas [17] describes for determining the number of clusters was used to interpret the concept map. However, no exact criteria are available for selecting the final number of clusters. The procedure began with an eight-cluster solution and successively increased or decreased the number of clusters by one. After each step, the different solutions were examined for their face value. A decision was made about whether in each solution the merger of clusters appeared to adequately represent the original data that the participants provided and whether it formed a coherent system of clusters. This procedure was continued until an illogical merger between two clusters, i.e., the statements did not share common content, appeared.

After determining the number of clusters, the participants’ ratings were reviewed. Two cluster-rating maps (figures) were constructed, one on the basis of relative *importance* and one on the basis of *predictive value*. Additionally, the data were analyzed separately for the four groups of participating healthcare service providers. In doing so, the mean rating for each cluster was calculated for each of the groups of participants.

### Ethical review

No formal ethical review by an institutional review board was necessary since the participants were healthy and were, as (trained) healthcare service providers, familiar with the proceedings of scientific research. Additionally, the study was not intrusive (two questionnaires to be completed on a website). Before the study started, each of the healthcare service providers who participated provided consent online after having been informed about the details of the study.

## Results

### Preparation

During the preparation stage, the focus prompt was formulated as “A factor that predicts whether a patient can be successfully transferred from SMHS to less intensive treatment is … “. The prompt specifically defined SMHS as specialist healthcare, but “less intensive treatment” was deliberately undefined in order to allow the various types of low-intensity mental-health services in the Netherlands and elsewhere to be considered. Examples of less intensive treatment are mental-health services provided by a general practitioner or low intensity (e.g., eight to twelve sessions per year) mental healthcare provided by a community mental-health nurse or a psychologist. Potential participants (*N* = 39) were invited to generate the statements anonymously. See Table 1 for participant characteristics (collected at step 3, the sorting / rating stage).

**Table 1:** Participant characteristics collected at step 3, the sorting / rating stage (*N* = 38).

Characteristic	Type of data	Data	Percentage
Gender	Female	31	81.6
Age	Range	22–54	
	Mean (SD)	38.1 (9.4)	
Occupation	General practitioner	7	18.4
	Peer worker	8	21.1
	Community mental health nurse	9	23.7
	Social worker	11	29.0
	Other	3	7.9
Experience (years)	Minimum-maximum	0–30	
	Median	10	
	Mean (SD)	13.7 (9.6)	

### Statements generated

Of the invited participants, 87% (*N* = 34) responded and completed the statement-generation stage of the study. In total, 54 statements were generated. After data cleaning, 31 statements remained, and these are shown in Table 2.

**Table 2:** Statements generated.

#	Statement (statement prompt: “A factor that predicts whether a patient can be successfully transferred from SMHS to less intensive treatment is … “)
1	The phase of recovery the patient is in.
2	The patient’s level of awareness of his illness.
3	The patient’s level of insight into his illness.
4	The patient’s motivation.
5	Whether the healthcare service provider who initiates the transfer has faith in the transfer.
6	Whether the patient gives consent.
7	Whether the patient’s general practitioner has faith in the transfer.
8	The quality of the patient’s support system.
9	The quality of the referral from one healthcare service provider to another.
10	The level of the patient’s self-reliance.
11	Whether the healthcare service providers have good cooperation.
12	Whether the patient’s support system has faith in the transfer.
13	Whether SMHS can be started quickly when needed.
14	Satisfying fulfillment of the patient’s social roles.
15	Whether the patient has a meaningful occupation.
16	The number of minutes of care the patient needs.
17	The number of people supporting the patient.
18	Whether the support given by healthcare service providers is adequate (not too little, not too much).
19	Whether the focus is on the patient’s opportunities.
20	Whether the patient is stable (no recent suicidal ideations or commitment to hospital).
21	Whether the patient has faith in the transition.
22	The patient’s hobbies.
23	Whether the help is aimed at controlling the patient.
24	How well the patient can formulate his request for help.
25	Whether the patient feels his life is meaningful.
26	The number of additional problems that person has, such as those related to work, living arrangements, or financial problems.
27	The patient’s learning capacity.
28	The degree of responsibilities the patient can bear.
29	Whether the patient has had successful experiences.
30	The patient’s skills.
31	Whether the focus is on the patient’s recovery and not on the symptoms.

### Structuring the statements

Potential participants (*N* = 57) were invited to participate in a second round of data collecting, with the aim of structuring the statements. They included the 39 participants from the previous step. Since the response rate from original participants in this second round of data collection was inadequate to reach the minimum *N* of 30 [15] in the sorting / rating stage (a separate data collection), additional potential participants were included according to the same procedures as the original participants in order to reach an adequate *N* (reaching a total *N* of 57 participants). The additional participants were needed because not all of the participants from Step Two (the first data collection) responded to the request to participate further. In fact, having different respondents in Step Two and Step Three of concept mapping is a common practice in concept mapping [17]. The participants (*N* = 38; 67%) that responded to the invitation in the third step began the sorting and rating tasks. Four participants either did not complete this step, or they provided unusable data; thus, 34 participants completed this step.

### Analysis and interpretation of the concept map

**Fig 1:**
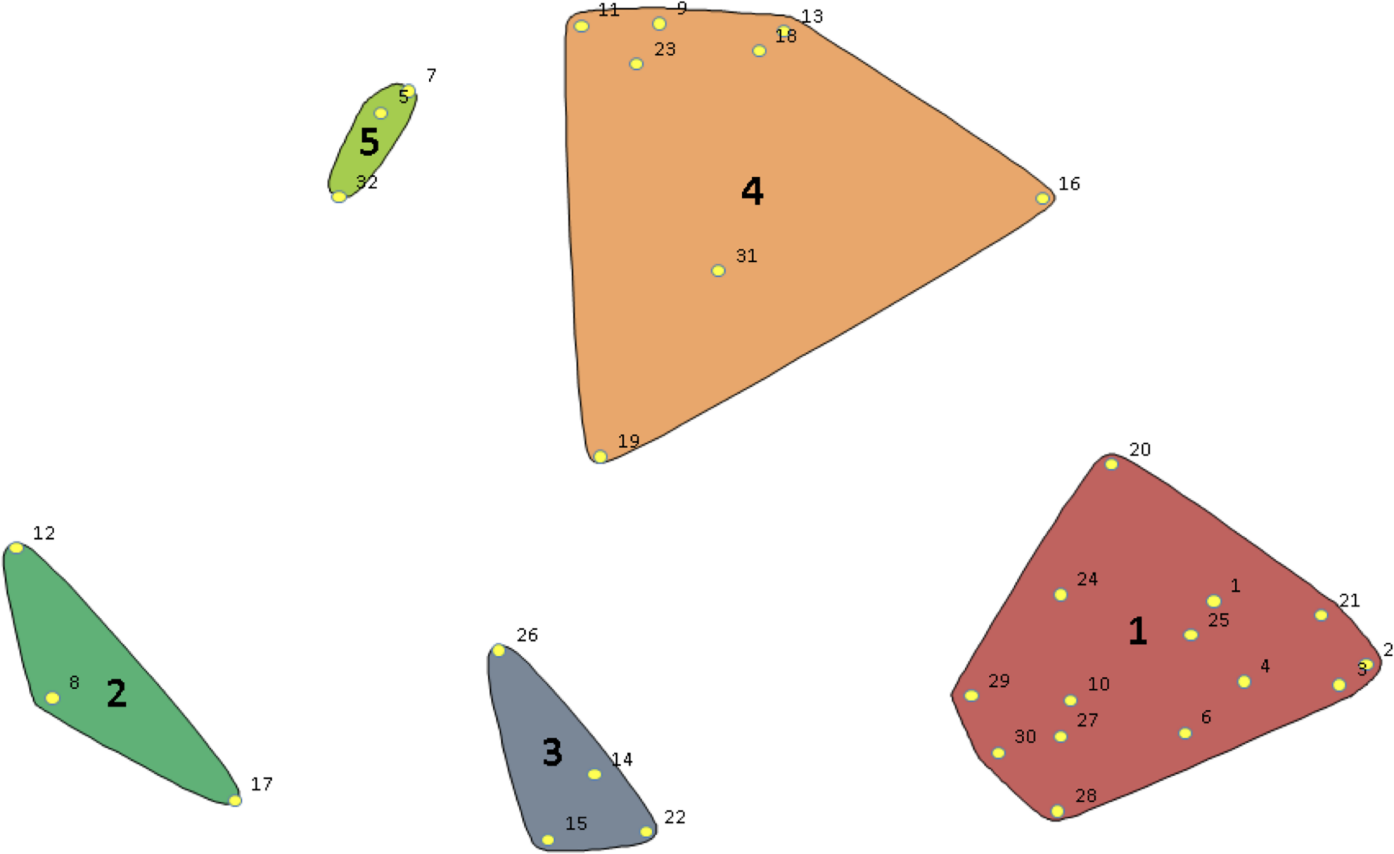
Map of the statements (in numbers) and their distances according to multi-dimensional scaling, visualized in the final clusters.

The analysis revealed a stress value of 0.18, which is well below the upper limit of 0.39, as identified by Rosas and Kane [16]. This means that there was sufficient stability in the data to proceed with the analysis of the concept mapping. In the fifth and final step, the statements were merged into a coherent model of clusters. Inspection of the different configurations indicated that a five-cluster solution provided the most detail and also yielded an extensive and coherent model (see Fig 1). For instance, going from a six-cluster to a five-cluster solution caused two clusters of patient-related statements to be merged; Statements 27 through 30 were now included in Cluster 1. Going from a five-cluster to a four-cluster solution caused Clusters 2 and 3 to be merged. This was judged to be illogical because Cluster 2 contained statements related to individual service providers, whereas Cluster 3 contained statements about the organization of services. In addition to the researchers, a convenience sample of three of the participating healthcare service providers was involved in the establishment of the concept map and the number of clusters.

### Themes in the clusters

The five clusters that emerged focus on the patient, the informal support system, the patient’s social situation, the organization of services, and the healthcare service providers. After the initial analysis and interpretation, ratings for each cluster were calculated for both importance and predictive value. The values for importance provided no additional insight into the predictive value of the importance ratings; therefore, these results are not included in this paper.

Cluster One includes *patient-related* statements. It is the cluster with the largest number of statements specifically about patients, such as their motivation, awareness of their illness, and their skills (see Table 3). According to participants’ ratings, this cluster has the greatest expected predictive value (5.54). Cluster Two was second highest on expected predictive value (5.26). It contains statements related to the *informal support system*. Along with Cluster Five, it is the smallest of the clusters. Cluster Three is focused on the patient’s *social situation*. Its expected predictive value (5.21) was higher than that of the clusters related to the healthcare service providers and the organization of services, but lower than the cluster related to the patient’s informal support system and the patient-related cluster. Cluster Four, the second largest cluster and the one with the second lowest expected predictive value (5.17), is centered on the *organization of services*. Cluster Five contains statements related to *healthcare service providers*, both the service providers in SMHS who make the referrals and the service providers in primary care who receive the referrals. It, along with Cluster Two, is the smallest of the clusters. It contains only three statements. The participants judged this cluster to have the lowest expected predictive value (5.12). When we inspect the individual items in the clusters, we see that four of the five highest scoring items on expected predictive value (Items 1, 4, 6, and 21) are in the cluster containing patient-related variables.

**Table 3:** Statements and corresponding ratings on expected predictive value.

Cluster (mean score of expected predictive value as scored by the participants)	Statement	Mean score of expected predictive value as scored by the participants
1: Patient-related (5.54)	Whether the patient has faith in the transfer.	6.25
	The patient’s motivation.	5.97
	The patient’s approval.	5.94
	The phase of recovery the patient is in.	5.94
	Whether the patient feels his life is meaningful.	5.76
	The patient’s skills.	5.67
	The patient’s level of self-reliance.	5.61
	Whether the patient is stable (no recent suicidal ideations or commitment to hospital).	5.58
	The patient’s level of awareness of his illness.	5.52
	The patient’s successful experiences.	5.52
	The patient’s level of insight into his illness.	5.48
	The degree of responsibility the patient can bear.	5.12
	The patient’s learning capacity.	4.97
	How well the patient can formulate his requests for help.	4.21
2: Informal Support System (5.26)	The quality of the patient’s support system.	5.67
	Whether the patient’s support system has faith in the transfer.	5.30
	The number of people supporting the patient.	4.82
3: Social Situation (5.21)	Satisfying fulfillment of the patient’s roles.	5.61
	The number of additional problems, such as work-related, living arrangements, or financial problems.	5.55
	Whether the patient has a meaningful occupation.	5.48
	The patient’s hobbies.	4.21
4: Organization of Services (5.17)	Whether the focus is on the patient’s opportunities.	5.79
	Whether SMHS can be started quickly when needed.	5.70
	Whether the support given by healthcare service providers is adequate (not too much, not too little).	5.64
	Whether the focus is on the recovery of the patient and not on the symptoms.	5.42
	The quality of the transfer between healthcare service providers.	5.39
	Whether the healthcare service providers have good cooperation.	5.15
	The number of minutes of care the patient needs.	4.48
	Whether the help is aimed at controlling the patient.	3.75
5: Healthcare service providers (5.12)	Whether the healthcare service provider can give the patient faith in the referral.	5.64
	Whether the healthcare service provider who initiates the transfer has faith in the transfer.	5.06
	Whether the patient’s general practitioner has faith in the transfer.	4.67

### Differences between groups of participants

When comparing the different groups of healthcare service providers, there are differences to be observed. These differences are limited due to the limited variation (the maximum variation is 0.67, 11% of the range of the likert-scale). All groups of participants except the general practitioners chose the patient-related cluster as the most important one and the one having the highest expected predictive value. In contrast, the general practitioners chose the patient-related cluster as the fourth highest of the five clusters on expected predictive value. The ordering of the clusters from highest expected predictive value to lowest expected predictive value is very similar for the social workers and the community mental-health nurses. The general practitioners, and to a lesser extent the peer workers, deviated from this order. The general practitioners chose the organization-of-services cluster as the one having the greatest expected predictive value. See Table 4 for additional results.

**Table 4:** Mean expected predictive value on each cluster as a function of healthcare service providers.

Cluster	General practitioners (N = 6)	Peer workers (N = 6)	Community mental health nurses (N = 9)	Social workers (N = 9)
1: Patient Related	4.99	5.55	5.61	5.66
2: Patients’ Informal Support System	5.14	4.90	5.15	5.50
3: Patients’ Social Situation	4.86	5.34	5.07	5.30
4: Organization of Services	5.31	5.47	5.22	5.18
5: Healthcare service providers	4.95	4.90	4.89	5.38

## Discussion

In this study, we explored the tacit knowledge of healthcare service providers that are involved in the care for people with SMI on factors that are expected to be predictive of successful referrals from SMHS to less intensive treatment. Five clusters with potential expected predictive value were identified: Patient-related factors, factors related to healthcare service providers, factors related to the organization of services, factors related to patients’ informal support system, and factors related to the patient’s social situation. The cluster related to patient-related factors was the largest cluster and the one rated as having the strongest expected predictive value. Factors related to the patient’s social situation and their informal support system were rated as the second and third highest on expected predictive value. Factors related to the healthcare service providers and the organization of services were rated as those having the lowest expected predictive value.

Factors related to patients and their informal social environment were viewed as having a higher expected predictive value than factors related to the service providers and the organization of health services. This is important information, because the focus on improving referrals is often aimed at the organization of care [18–20]. Targeting factors related to patients instead of the factors related to the organization of care might be essential for improving the success of referrals from SMHS to less intensive treatment. In practice, this means that it might be more effective to invest in the needs of individual patients and their informal support system, than to start a project to improve collaboration with general practitioners. This issue is worthy of further research, as it might have considerable consequences.

When the data were analyzed according to the occupation of the healthcare service providers, potential differences can be observed. Although the the differences between the service provider’s disciplines are small, the participants appear to fall into two groups. The community mental-health nurses had views similar to those of the social workers. The general practitioners had similar views to the peer workers, as seen in every cluster except cluster five (healthcare service providers). This difference in views between the service provider’s disciplines could complicate collaboration, since diverging views may result in different decisions, an effect seen more frequently in cross-disciplinary healthcare collaboration [21–22]. Knowing this, establishing a common view is essential in a collaboration between different service provider’s disciplines.

### Strengths and limitations

There are some limitations of this study. For instance, there was a skewed distribution of participants’ gender; 81.6% of the participants were female. This, however, is in line with the mean percentage of females working in healthcare in the Netherlands (79.4%) [23]. Another limitation of the study is that it sampled participants only from the Netherlands. An international sample would have allowed the results to be more generalizable because such a study would have been conducted in multiple healthcare systems. This, however, would also have reduced the clarity of the results. This study might also have benefitted from a more diverse sample of participants, including for instance patients and their significant others, and possibly other healthcare service providers (e.g. psychologists, psychiatrists and nurses from SMHS).

The study was conducted using a concept mapping design. Concept mapping has been developed into a reliable and valid research method [16] that has become increasingly popular [24, 25]. The concept-mapping method was appropriate for the present research question because it allowed us to efficiently explicate, organize, and rate tacit knowledge on the topic being investigated. However, the concepts of ‘importance’ and ‘predictive value’ appeared closesely related, a more diverging concept (e.g. difficulty or feasibility) could have provided additional insights. The study was sufficiently large (*N* = 34), when compared to standard sample sizes of 20 to 30 participants [16]. When the participants are divided into subgroups, the sample sizes become small and differences between these subgroups have limited reliability. A larger sample size would possibly have provided stronger results and further research is thus warranted. The use of an online concept mapping approach is a strength of this study, as it extends the methodology and it allowed for a broad and geographically diverse sample, that would not have been possible using traditional (offline) concept mapping approaches. Finally, the method of concept mapping allowed the factors and clusters to be arranged in a hierarchy, and this creates an opportunity for future research on this topic to focus on the factors with the strongest expected predictive value.

### Comparison with existing knowledge

The five clusters identified in the study partially overlap with the clusters and topics in the Threshold Assessment Grid. The Threshold Assessment Grid is an instrument that was developed to improve the quality and quantity of referrals from general healthcare to SMHS [26–31] It contains three clusters of items on (a) safety, (b) risks, and (c) needs and disabilities. Most of the items and clusters identified in the present study match the items in the needs and disabilities cluster of the Threshold Assessment Grid. The main difference between the Threshold Assessment Grid and the current study lie in the importance of the social support system. Factors considering social support are absent in the Threshold Assessment Grid, but present in the current study.

Differences between the content of the Threshold Assessment Grid and that of the present study can be accounted for by the fact that there were different participants in the two studies. Participants in the Threshold Assessment Grid included general practitioners and healthcare service providers from SMHS, whereas the present study included a broader sample of healthcare service providers from general healthcare services. Because the two studies included different groups of participants, the results from the two studies complement each other. Although patient-related factors were found in both studies, the Threshold Assessment Grid had a narrower sample of healthcare service providers than did the present study, and it had a major focus on factors related to safety and risks [32]. By contrast, the present study included a broader selection of healthcare service providers from general healthcare, and it included additional factors, such as those related to social-support systems, healthcare service providers, and the organization of services.

The present study substantially overlaps with the CHIME-framework [33] for personal recovery. This is significant because recovery-oriented services are associated with a higher rate of successful referrals [34], and it thus validates the results of the present study, which is related to the personal recovery of the patients. The CHIME-framework (which includes factors related to connectedness; hope and optimism about the future; identity; meaning of life; and empowerment) is a conceptual framework of personal recovery, which was derived from the results of 97 studies that had been conducted up until 2011. Most of the recovery processes identified in the CHIME-framework are also reflected in the patient-related cluster in the present study. Examples of these processes are: hope and optimism about the future, personal identity, meaning of life, and empowerment. An exception can be found in *connectedness*, which is the first process in the CHIME-framework. It coincides with the cluster related to informal support systems, and it partly coincides with the cluster related to patients’ social situation, which was found in the present study. It is not unexpected that the other two clusters found in the present study are not reflected in the CHIME-framework. This difference occurs because the CHIME-framework is strictly patient-related, whereas the present study was not.

The lack of sufficient information about which factors best predict successful referrals from SMHS to less intensive treatment is a problem that has consequences for patients, service providers in healthcare services, and financial providers of healthcare services. To date, no study has identified the complete range of factors that affect successful referrals from SMHS to less intensive treatment. The results of the present study do not provide the clear-cut criteria that are needed for predicting successful referrals from SMHS to less intensive treatment. However, combined with the results of earlier studies, this study provides a basis for further research. Our results mainly suggest that, in addition to factors earlier identified (mostly patient-related factors, safety and risks), the social support system should be subject of further study. Both the service provider and the informal support of the patient might have a previously overlooked but important influence on the referral from SMHS to less intensive treatment and should be further explored, preferably with a prospective, quantitative design. When we understand the influence of the social support system and other factors, we can improve the procedures for referring patients with SMI from SMHS to less intensive treatment.

## Conclusions

Knowledge is limited both about which factors are predictive of successful referrals and what the views are of different service providers who are involved. The present study helps to clarify these factors, and it also provides a basis for future research. The results of the present study confirm the importance of patient-related factors and factors related to patients’ social situation that were identified in earlier studies. The study also identified additional factors—including patients’ informal support system, the organization of services, and the views of different healthcare service providers—that should be taken into account when patients are being discharged from SMHS.
